# Psychometric properties of the kidney disease quality of life-36 (KDQOL-36) in Ethiopian patients undergoing hemodialysis

**DOI:** 10.1186/s12955-022-01932-y

**Published:** 2022-02-10

**Authors:** Mignote Hailu Gebrie, Hussen Mekonnen Asfaw, Workagegnehu Hailu Bilchut, Helena Lindgren, Lena Wettergren

**Affiliations:** 1 College of Medicine and Health Sciences, School of Nursing, University of Gondar, Gondar, Ethiopia; 2 College of Health Sciences, School of Nursing & Midwifery, Department of Nursing, Addis Ababa University, Addis Ababa, Ethiopia; 3 College of Medicine and Health Sciences, School of Medicine, Department of Internal Medicine, University of Gondar, Gondar, Ethiopia; 4 Department of Women’s and Children’s Health, Division of Reproductive Health, Karolinska Institutet, Solna, Sweden; 5 Department of Public Health and Caring Sciences, Uppsala University, Uppsala, Sweden

**Keywords:** Confirmatory factor analysis, End stage renal disease, KDQOL-36, Quality of life, Reliability, Validity, Hemodialysis, Ethiopia

## Abstract

**Background:**

Health-related quality of life (HRQOL) has a direct association with increased morbidity and mortality among end stage renal disease patients. Valid and reliable instruments to measure the HRQOL of patients with end stage renal disease are therefore required. This study aimed to translate, culturally adapt and evaluate the psychometric properties of the Amharic version of the Kidney Disease Quality of Life-36 (KDQOL-36) instrument in Ethiopian patients with end stage renal disease undergoing hemodialysis.

**Methods:**

The KDQOL-36 instrument was developed for individuals with kidney disease who are being treated with dialysis and includes both generic and disease-specific components. The KDQOL-36 was translated to Amharic language and distributed to a cross-sectional sample of 292 hemodialysis patients. The psychometric evaluation included construct validity through corrected item-total correlation, confirmatory factor analysis and known group analysis. Convergent validity was evaluated by correlations between each of the three kidney disease targeted scales (symptoms/problems list, burden of kidney disease and effects of kidney diseases) and the European Quality of Life 5D-5L and Visual Analog Scales. Regarding reliability, internal consistency and test–retest reliability were assessed.

**Results:**

Two hundred ninety-two patients with a mean age of 48 (SD ± 14.7) completed the questionnaire. Corrected item- total correlation scores were > 0.4 for all items. Confirmatory factor analysis revealed a two *χ*^2^ /df was 4.4, Root Mean Square Error of Approximation (RMSEA) = 0.108 (90% CI 0.064–0.095), Comparative Fit Index (CFI) = 0.922, Tucker Lewis Index (TLI) = 0.948 and Standardized Root mean-squared residual (SRMR) = 0.058) and three *χ*^2^ /df = 3.1, RMSEA = 0.085 (90% CI 0.064–0.095), CFI = 0.854, TLI = 0.838 and SRMR = 0.067) factor models for the generic and disease specific components respectively. The mean scores of the three kidney disease targeted domains were correlated to the EQ-5D-5L & VAS with correlation coefficients of large magnitude (0.55–0.81). The reliability of the instrument was satisfactory (Cronbach’s alpha = 0.81–0.91) and Intra-class correlation (ICC) = 0.90–0.96).

**Conclusion:**

The Amharic version of the KDQOL-36 is a reliable and valid instrument recommended for assessment of HRQOL of Ethiopian patients on hemodialysis.

**Supplementary Information:**

The online version contains supplementary material available at 10.1186/s12955-022-01932-y.

## Background

End stage renal disease (ESRD) is a public health problem resulting in high morbidity and mortality worldwide [1–4]. Globally in 2017, chronic kidney disease (CKD) resulted in 1·2 million deaths, more than did tuberculosis or Human Immunodeficiency Virus (HIV) [5]. It is the terminal phase of CKD, where the kidneys experience complete or near complete failure and renal replacement therapy is needed to sustain life [6–8].

The worldwide prevalence of ESRD ranges from 2447 cases patients per million population (pmp) in Taiwan to 10 cases pmp in Nigeria. Much less is known in all of Africa, due to lack of renal registry, with the highest prevalence of ESRD in Tunisia (713 pmp) and Egypt (669 pmp). However, it is predicted that the low-income countries of Asia and Africa will be where more than 70% of patients with ESRD will live by the year 2030 [9]. In Ethiopia, the incidence of CKD is increasing as a result of increasing prevalence of CKD risk factors such as hypertension and diabetes mellitus [10].

Hemodialysis is one of the treatment methods, contributing to increased survival in patients with ESRD [11, 12]. However, patients with hemodialysis suffer from a multitude of problems including sleep disorders, peripheral neuropathy, infection, fatigue, stress, anxiety, depression, cognitive difficulties pain and sexual dysfunction [13–15]. Hence, assessing the health-quality of life (HRQOL) of ESRD patients is essential as it is an independent predictor of patient’s treatment outcome.

Several instruments have been developed to measure HRQOL in ESRD patients [16–18]. The kidney disease quality of life (KDQOL-36) survey is a disease-specific measure of HRQOL including both generic and disease-specific components. The KDQOL-36 contains 5 subscales and is one of the most commonly used measures for assessment of patients with kidney disease [16, 19]. The instrument has been translated and validated in various languages [20–23], however, to date its psychometric properties have not been confirmed in an Ethiopia population. Therefore, the objective of this study was to translate, culturally adapt and evaluate the psychometric properties of the KDQOL-36 questionnaire when used among Ethiopian patients with ESRD undergoing hemodialysis.

## Methods

### The KDQOL-36 instrument

The KDQOL-36 was derived from the original 134-item KDQOL instrument [24]. The KDQOL-36 version includes the Medical Outcomes Study’s 12-Item Short-Form Health Survey (SF-12) as a generic core and the 24-item kidney disease targeted questionnaire [25, 26]. The items of the SF-12 are summarized into the Physical Component Summary (PCS) score and the Mental Component Summary (MCS) with response alternatives varying from 2- to 6-point scales [27, 28]. The kidney disease targeted instrument includes three scales: Symptoms and Problems (12 items), Burden of Kidney Disease (4 items), and Effects of Kidney Disease (8 items); all items have 5 response options. The scale scores of the KDQOL-36 questionnaire (PCS, MCS, symptoms and problems, burden of kidney disease, effects of kidney disease,) are transformed to 0 to 100 with higher scores indicating better HRQOL [24, 29]. We used the KDQOL^Tm^-36 scoring program (V 2.0) from the University of California, Los Angeles (UCLA) to compute the scale scores. This program is available free for download online (http://www.rand.org/health/surveys_tools/kdqol.html).

### Translation of the KDQOL-36 into amharic language

The KDQOL-36 was translated from English into Amharic by two professional translators (native Amharic speakers with fluency in English). The translators had a Master’s degree in English Language and Literature. The translated Amharic versions were then reviewed by a committee of experts including the investigators, the original translators, nurses working in a hemodialysis unit, a nephrologist and experts in instrument development and translation. Some minor changes were made before the items were translated back into English by two other independent professional translators and were then compared to the original instrument. There were repeated back translations for some items for which deviations were encountered until matching was seen to be sufficiently good to ensure that the Amharic version did not differ from the original instrument. Finally, cognitive testing was conducted on 10 ESRD patients undergoing hemodialysis to determine its cultural appropriateness and acceptability including instructions, items and response choices [29] which resulted in rewording a few items. Translation of the English KDQOL-36 to Amharic was performed according to the basic guidelines for translating surveys (see https://www.rand.org/health-care/surveys_tools/about_translations.html).

### Sample and settings

A cross-sectional study was conducted at two governments (Menelik II and Zewditu memorial) and six private (Ethio-tebib, Hallelujah, Hayat, Bethel, MABD and Flow) hospitals/dialysis centers situated in Addis Ababa, Ethiopia. Data for the psychometric evaluation were collected over a period of one month, January to February 2021, from patients receiving outpatient maintenance hemodialysis. All patients who fulfilled the inclusion criteria (ESRD patients aged ≥ 18 years, maintained on regular hemodialysis treatment for ≥ 3 months and speaking Amharic) were approached regarding possible participation in the study. Hemodialysis patients who provided their consent were then interviewed by trained research assistants and their medical records were consulted to obtain clinical data. Participants were asked to respond to study-specific and standardized items (KDQOL-36). Two weeks later, a subsample (n = 50) was asked to respond to the Amharic KDQOL-36 again to assess the test–retest reliability.

### Additional measures

In addition to the KDQOL-36, participants were asked to complete the Amharic European Quality of Life 5D-5L and Visual Analog Scale (EQ-VAS) [30]. The EQ-5D-5L is a generic instrument, developed by the European quality of life (EuroQol) Group consisting of five dimensions: mobility, self-care, usual activities, pain/discomfort, and anxiety/depression and an EQ-VAS with a 5-point Likert scale (no problems, slight, moderate, severe, extreme/unable). The EQ-VAS is numbered from 0 to 100, where 0 indicated the worst imaginable health and 100 was best imaginable health [31]. Scores were converted to 0 to 100%.

### Statistical analysis

STATA version 14 was used for statistical analysis [32]. Normality was assessed for the outcome variables using the Shapiro Wilk test. Patients’ demographic and clinical characteristics were summarized using descriptive statistics (percentages, frequencies, means, standard deviations). Descriptive statistics for the five separate domains of the KDQOL-36 were calculated with means and standard deviation [29].

### Data quality

Data quality was assessed by examination of missing values for each item of the KDQOL-36. Furthermore, we evaluated whether all response alternatives were used for all items as well as floor and ceiling effects. Ceiling effect was measured by the proportion of people rating the highest possible score while floor effects were measured by the proportion of people rating the lowest possible score. These effects were considered significant if > 15% of the patients scored the lower/higher values [32].

### Construct validity

Construct validity of KDQOL-36 was assessed by using corrected item scale correlation using cut-off scores ≥ 0.4 to indicate adequate correlation [33]. Confirmatory factor analysis (CFA) was employed to test the model fit between the observed and the hypothetical measures. CFA is a method of choice when the researcher has prior knowledge of the basic latent variable construction [34]. The SF-12 and the kidney disease targeted questionnaire were analyzed separately because they are different questionnaires each having their own unique contribution to the assessment of HRQOL. The validity of SF-12 health survey would be supported if the hypothesized physical and mental component summary scales were identified [27]. CFA would support the disease-targeted part if a three-factor structure was achieved [29]. Model fit was assessed using normed Chi-Square (χ2/df), Root Mean Square Error of Approximation (RMSEA), Comparative Fit Index (CFI), Tucker Lewis index (TLI) and Standardized Root mean-squared residual (SRMR). Normed Chi-Square should be 0–2 for good fit and ≤ 3 for an acceptable fit. Values between 0.05 and 0.08 suggest reasonable RMSEA and lower values represent better fit [35]. CFI & TLI > 0.9 indicate good fit and that SRMR should be < 1.0 to consider the model is favorable [36]. We used Maximum likelihood estimation (MLE) [34].

Convergent validity was evaluated by correlation coefficients between the three kidney disease targeted scales with the EQ-5D-5L and EQ-VAS. Pearson’s correlation coefficient was calculated where values 0.10–0.29, 0.30–0.49 and > 0.49 represents small, moderate and large magnitude, respectively [37]**.** It was hypothesized that the three kidney disease specific domains would have moderate to large coefficients with the ED-5D-5L and VAS, respectively.

Known group analyses were conducted to test how well the questionnaire discriminates between subgroups of the study sample that differed in diabetes status, examining a hypothesis supported by previous research [38, 39]. It was expected that patients without diabetes would have better HRQOL than diabetic patients. Independent t-tests and an analysis of variance (ANOVA) were used to evaluate the differences.

### Reliability

Reliability included internal consistency as well as test–retest. Internal consistency was estimated using Cronbach alpha coefficient for the different domains of the instrument. Cronbach’s alpha between 0.70–0.90 is suggested to reflect adequate internal consistency [40]. Intra-class correlation coefficient (ICC) was computed to assess test–retest reliability where values > 0.9, 0.75–0.90, 0.5–0.75 and < 0.5 indicates excellent, good, moderate and poor reliability respectively [41].

## Results

### Socio-demographic characteristics

The total sample included 292 patients (response rate 96%) on maintenance hemodialysis with a mean age 48 (SD ± 14.7), please see Table 1.

**Table 1:** Socio-demographic characteristics of patients with ESRD undergoing hemodialysis in Addis Ababa, Ethiopia, 2021 (n = 292)

Variables	Category	Numbers (n)	Percent (%)
Sex	Male	187	64
	Female	105	36
Age (years), M (SD)	48 (SD ± 14.7)		
Residence	In Addis Ababa	250	85.6
	Outside Addis Ababa	42	14.4
Marital status	Ever married	232	79.5
	Single	60	20.5
Educational status	Not read and write	26	8.9
	Read and write	29	9.9
	Primary (1–8)	33	11.3
	Secondary (9–10)	32	11.0
	Preparatory (11–12)	52	17.8
	Vocational	10	3.4
	Diploma and above	110	37.7
Occupation	Employed	152	52.1
	Unemployed	140	47.9
Hemodialysis session per week	1 times	15	5.1
	2 times	125	42.8
	3 times	152	52.1
Funding	Yes	70	24
	No	222	76
Family history	Yes	11	3.8
	No	281	96.2
Duration since start of hemodialysis, M (SD)	2.4 years (SD ± 2.1)		
Vascular access type	Arteriovenous fistula	218	74.7
	Arteriovenous graft	30	10.3
	Permanent catheter	31	10.6
	Temporary catheter	13	4.5

*M* mean; *SD* standard deviation

### Data quality

There were no missing data. All response alternatives were used for all items. Descriptive statistics including floor and ceiling effects are shown in Table 2.

**Table 2:** Descriptive statistics for the KDQOL-36 questionnaire among hemodialysis patients in Addis Ababa, Ethiopia, 2021 (n = 292)

Domains	No. of items	Mean (SD)	Range of items mean (SD)	Floor/ceiling effects, %	Cronbach’s alpha
*Kidney disease targeted scales*					
Symptoms/problems list	12	68.19 (19.52)	57.10–76.28(24.5–29.8)	0/1.0	0.907
Effects of kidney disease	8	57.13 (18.28)	39.29–66.44(22.1–30.9)	0/0	0.827
Burden of kidney disease	4	35.57 (29.82)	25.43–44.35(30.9–38.6)	10.6/2.7	0.899
*12-item health survey (SF-12)*					
SF-12 PCS	6	35.59 (8.80)	24.32–43.49(29.8–47.1)	0/0	0.813
SF-12 MCS	6	38.85 (13.63)	38.56–47.05(28.6–47.1)	0/0	0.892

### Construct validity

**Fig. 1:**
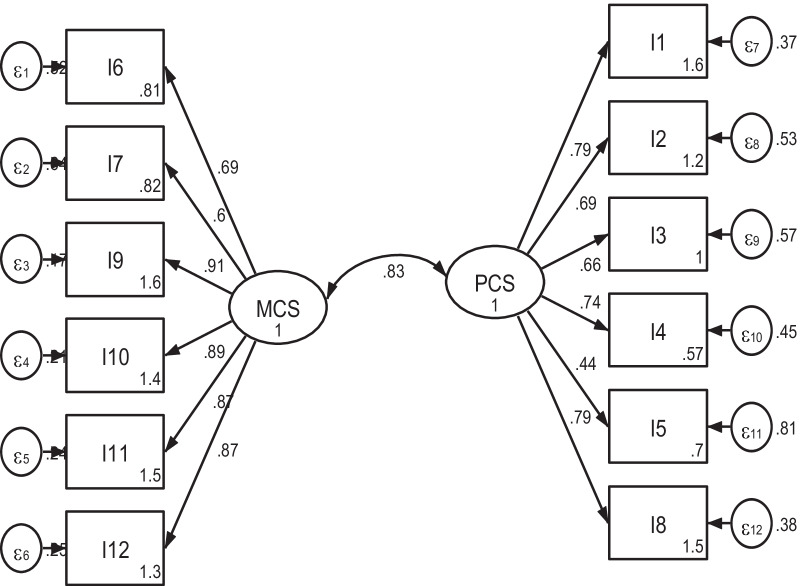
A two-factor model for the SF-12 PCS & MCS scales from confirmatory factor analysis among hemodialysis patients in Addis Ababa, Ethiopia, 2021

**Fig. 2:**
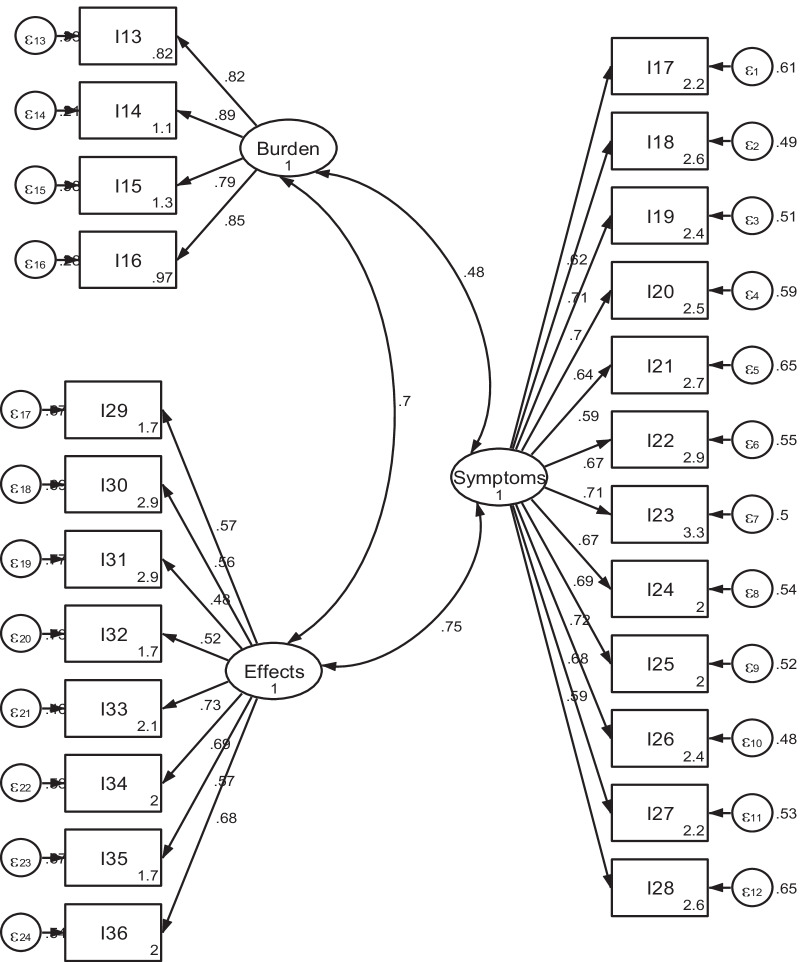
A three-factor model for the disease-targeted scales obtained from confirmatory factor analysis among hemodialysis patients in Addis Ababa, Ethiopia, 2021

The corrected item-total correlation coefficients for all items were between 0.41 to 0.85 (data not shown), thus showing adequate correlation. The CFA results for SF-12 showed that *χ*^2^/df was 4.4, RMSEA = 0.108 (90% CI 0.064–0.095), CFI = 0.922, TLI = 0.948 and SRMR = 0.058 (Fig. 1). Since the RMSEA was relatively high, we re-specified the model (supplementary file 1). The model outputs were similar and as the uncorrected model fitted well with the originally hypothesized two-factor model which forms the basis of the PCS and MCS this was selected. Similarly, in the kidney disease related scales (symptoms/ problems, effects and burden), the data fitted with the hypothetical three factor model with *χ*^2^/df = 3.1, RMSEA = 0.085 (90% CI 0.064–0.095), CFI = 0.854, TLI = 0.838 and SRMR = 0.067) (Fig. 2). Model re-specification was considered since the RMSEA & SRMR were a bit high and the CFI & TLI were a bit low. Even though some variability was found that the data couldn’t explain, the results were very close to the uncorrected model (supplementary file 2), and we decided to use the uncorrected one. Thus, the kidney disease targeted parts of the KDQOL-36 instrument was revealed as a three factor model as it was hypothesized. The factor loadings of all items in both parts (SF-12 and diseases specific) were positive and exceeded the threshold of 0.4, which indicates considerable interpretability of original factor structure.

The associations between the three kidney-disease specific domains and the EQ-5D-5L and the EQ-VAS are presented in Table 3. Moderate to strong correlations were evidenced between KDQOL sub scales and the EQ-5D-5L as well as the sub scales and VAS.

**Table 3:** Correlation between the domains of the Amharic version of the KDQOL-36 and the EQ-5D-5L and the VAS among hemodialysis patients in Addis Ababa, Ethiopia, 2021 (n = 292)

Domains	No. of items	VAS correlation (r) with *P* value < 0.0001	EQ-5D-5L correlation (r) with *P* value < 0.0001
*Kidney disease targeted scales*			
Symptoms/problems list	12	0.585	0.683
Effects of kidney disease	8	0.694	0.803
Burden of kidney disease	4	0.548	0.603

Regarding known group validity, patients with diabetes had significantly lower scores for the effects of kidney disease, SF-12 PCS and SF-12 MCS scales (Table 4).

**Table 4:** Differences on scale scores of the KDQOL-36 among hemodialysis patients in Addis Ababa, Ethiopia, 2021

Variables	KDQOL-36 sub scales (mean, SD)				
	Symptoms/problems list	Effects of kidney disease	Burden of kidney disease	SF-12 PCS	SF-12 MCS
*Diagnosis of diabetes*					
Yes (103)	73.2	50.7	28.1	33.9	35.5
No (188)	76.1	56.8	29.8	36.3	40.6
*P* value	0.144	0.015*	0.566	0.024*	0.006*

*Statistically significant

### Reliability

Cronbach’s alpha values for the five subscales exceeded 0.80 (Table 2). With regard to test–retest reliability, the subscales had an ICC value ranging between 0.90 to 0.96 (data not shown).

## Discussion

In this study, we evaluated the psychometric properties of the translated and culturally adapted Amharic version of KDQOL-36. Our results indicate that the Amharic version of the KDQOL-36 with the given response alternatives is satisfactory for use among patients who understand the Amharic language. Furthermore, the floor and ceiling effects of the Amharic version of the KDQOL-36 instrument were minimal which is in line with an American study [42]. However, it contrasts with findings from another study in the USA which found a significant floor effect in the ‘burden of kidney disease’ and ‘effects of kidney disease’ subscales [22]. The difference in findings could be explained by the reason why psychometric testing of self-report measures is required for use in different languages and on patients from different cultural backgrounds [43]. The minimal floor/ceiling effects indicate that the scales can accurately measure the concept by capturing the deterioration or improvement in the course of the disease along with hemodialysis therapy.

All correlation coefficients in the corrected item-total correlation testing of the Amharic version of the KDQOL-36 were above 0.4 confirming construct validity of the Amharic version instrument. In line with the previous study in Singapore [23], the United States [42], China [44] and Malaysia [45], our data fit the three factor structure (symptoms/ problems list, burden of kidney disease and effects of kidney disease on kidney life). For the 12-item health survey, we found that a two factor structure (PCS and MCS) as we had hypothesized based on the SF-12 item health survey construction [27]. The factor loadings of all items in the disease-targeted scales and SF-12 health survey exceeded 0.4.

The correlations of the three kidney disease targeted scales with the EQ-5D-5L and EQ-VAS were all in the expected direction and the coefficients were of large magnitude supporting convergent validity of these scales. This finding was comparable with results from previous studies [20, 45]. The Amharic version of the KDQOL-36 established evidence of known group validity as the scale scores were able to discriminate between subgroups of patients in relation to diabetes status. In this study, patients with diabetes had a worse quality of life than those without diabetes as expected and the reason could be complications resulted from the disease. This finding was supported by other recent studies evaluating the quality of life of ESRD patients [38, 39, 42, 46].

In our study, the three kidney disease targeted scales showed good internal consistency (Cronbach’s alpha > 0.8) in accordance with previous studies conducted in China [44], Malaysia [45], Thailand [20], Korea [47] and the United States [22, 42]. Similarly, the generic part of the instrument showed good internal reliability, which was comparable to previous studies in Malaysia [45], Iran [48], India [49] and Singapore [23]. At two-weeks test–retest, the ICC value ranged 0.090–0.961 for all the five scales, which was consistent with previous studies [20, 49, 50]. Thus, the Cronbach’s alpha and ICC values suggested that the Amharic version of KDQOL-36 instrument is reliable.

### Strengths and limitations

This study has several strengths. First, with a large sample (response rate 96%) including patients from government as well as private hospitals, our sample was representative of the population undergoing hemodialysis in Addis Ababa, Ethiopia. Second, sample size of approximately 300 patients enabled rigorous statistical analyses to evaluate validity (construct and known-groups validity). There are also a few limitations to be considered when concluding our results. Amharic is the most commonly used language in the country. Still, two patients were excluded as they did not speak or understand Amharic, and if they represent a subgroup that may perceive the items of the questionnaire in a different way is not known. The study collected data by face-to-face interviews though self-administration, a procedure commonly practiced in administering the KDQOL-36 instrument. We choose this mode of administration as considerably large proportion of the population in Ethiopia cannot read and write and participants in pilot interviews expressed that they preferred to be interviewed in front of instead of filling out the questionnaire by themselves. It should also be noted that a higher proportion of the study participants was well educated compared to the population in the country as a whole. However, this reflects the actual population receiving hemodialysis due to kidney disease and not to be considered a selection bias. Additionally, despite the fact that we recruited 292 patients, the test–retest analysis was based on a smaller convenient portion of this group (50 patients). This could possibly inject a selection bias in to our test–retest analysis and skew the results. However, when compared to the rest of the cohort, it exhibited no significant differences in demographic variables.

## Conclusion

The KDQOL-36 in the Amharic language appears to be valid and reliable for measuring HRQOL of Ethiopian patients with end stage renal disease undergoing hemodialysis. The measure is recommended for use in clinical research in Ethiopia.

## Supplementary Information

**Additional file 1:** A re-specified two-factor model for the SF-12 PCS & MCS scales from confirmatory factor analysis among hemodialysis patients in Addis Ababa, Ethiopia, 2021.

**Additional file 2:** A re-specified three-factor model for the disease-targeted scales obtained from confirmatory factor analysis among hemodialysis patients in Addis Ababa, Ethiopia, 2021.

## Data Availability

The datasets generated and/or analyzed during the current study are available from the corresponding author on reasonable request.

## Abbreviations

CKD: Chronic kidney disease
CFA: Confirmatory factor analysis
EQ-VAS: European quality of life visual analog scale
ESRD: End stage renal disease
HRQOL: Health-related quality of life
ICC: Intra-class correlation
KDQOL-36: Kidney disease quality of life-36
MCS: Mental component summary
PCS: Physical component summary
SF-12: Short form-12
QOL: Quality of life
