# Supplementary figures and images for: Psychometric properties of the kidney disease quality of life-36 (KDQOL-36) in Ethiopian patients undergoing hemodialysis
Source: Health Qual Life Outcomes. 2022 Feb 10;20:24. doi: 10.1186/s12955-022-01932-y (PMC8832803; doi:10.1186/s12955-022-01932-y)

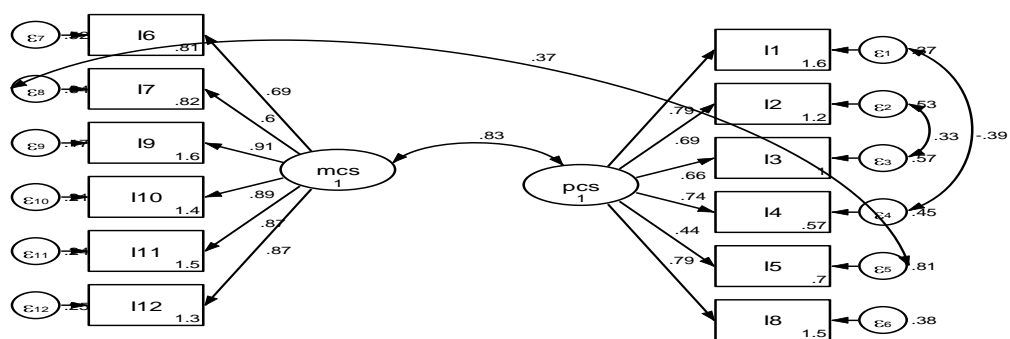

Supplement: Supplementary file 1 — Additional file 1: A re-specified two-factor model for the SF-12 PCS & MCS scales from confirmatory factor analysis among hemodialysis patients in Addis Ababa, Ethiopia, 2021. [file 12955_2022_1932_MOESM1_ESM.pdf]

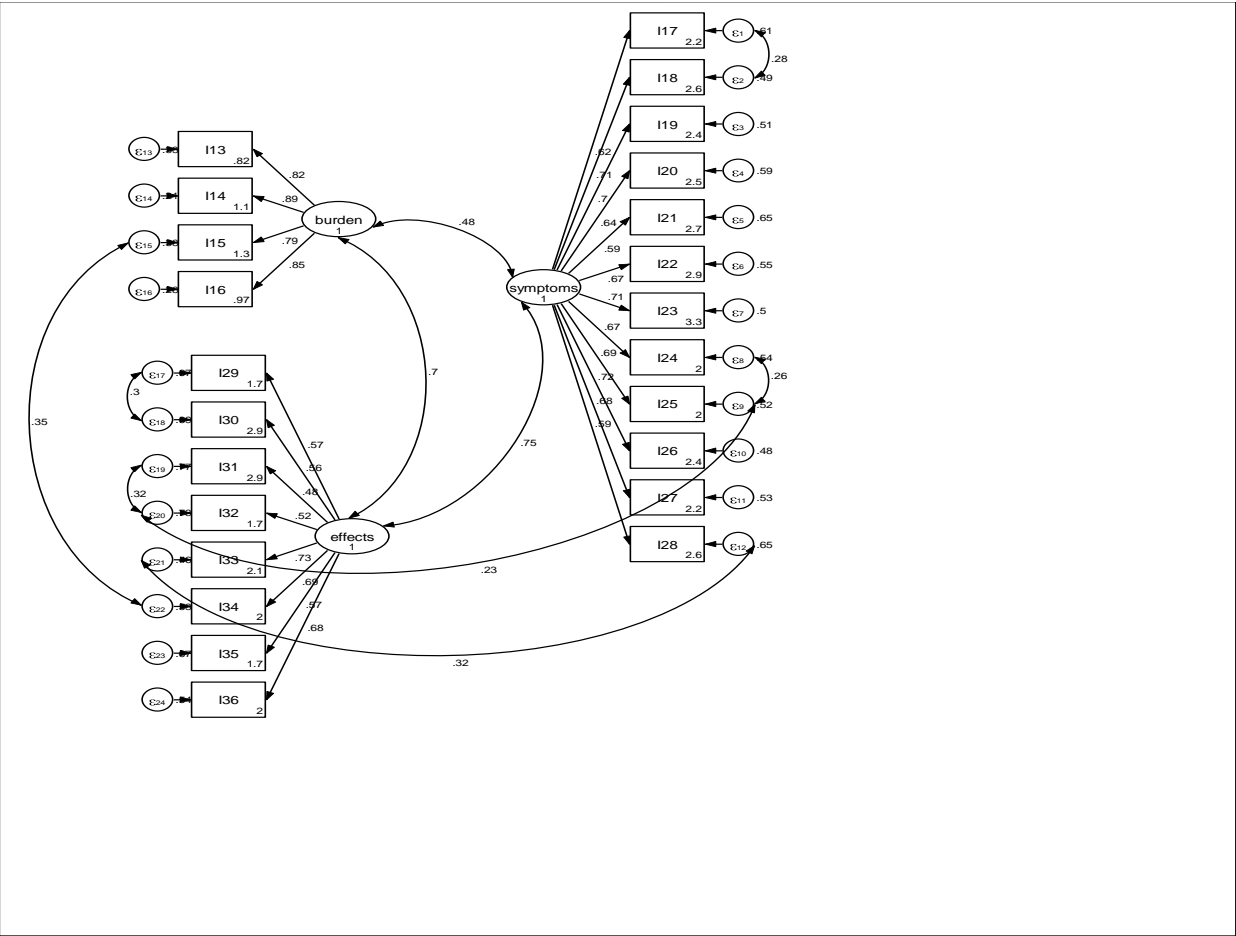

Supplement: Supplementary file 2 — Additional file 2: A re-specified three-factor model for the disease-targeted scales obtained from confirmatory factor analysis among hemodialysis patients in Addis Ababa, Ethiopia, 2021. [file 12955_2022_1932_MOESM2_ESM.pdf]
